# A Well-Established Gut Microbiota Enhances the Efficiency of Nutrient Metabolism and Improves the Growth Performance of *Trachinotus ovatus*

**DOI:** 10.3390/ijms25105525

**Published:** 2024-05-18

**Authors:** Miao Kong, Wendong Zhao, Cong Wang, Jie Qi, Jinxiang Liu, Quanqi Zhang

**Affiliations:** 1 Key Laboratory of Tropical Aquatic Germplasm of Hainan Province, Sanya Oceanographic Institution, Ocean University of China, Sanya 572025, China; km17669309286@163.com (M.K.); wendongzhaoa@163.com (W.Z.); wangcong11011@stu.ouc.edu.cn (C.W.); qijie@ouc.edu.cn (J.Q.); liujinxiang@ouc.edu.cn (J.L.); 2 MOE Key Laboratory of Marine Genetics and Breeding, Ministry of Education, Ocean University of China, Qingdao 266003, China

**Keywords:** gut microbiota, growth, antibiotic-treated, multiomics analysis, *Trachinotus ovatus*

## Abstract

The gut microbiota has become an essential component of the host organism and plays a crucial role in the host immune system, metabolism, and physiology. Nevertheless, our comprehension of how the fish gut microbiota contributes to enhancing nutrient utilization in the diet and improving host growth performance remains unclear. In this study, we employed a comprehensive analysis of the microbiome, metabolome, and transcriptome to analyze intestines of the normal control group and the antibiotic-treated model group of *T. ovatus* to investigate how the gut microbiota enhances fish growth performance and uncover the underlying mechanisms. First, we found that the growth performance of the control group was significantly higher than that of the antibiotic-treated model under the same feeding conditions. Subsequent multiomics analyses showed that the gut microbiota can improve its own composition by mediating the colonization of some probiotics represented by *Lactobacillus* in the intestine, improving host metabolic efficiency with proteins and lipids, and also influencing the expression of genes in signaling pathways related to cell proliferation, which together contribute to the improved growth performance of *T. ovatus*. Our results demonstrated the important contribution of gut microbiota and its underlying molecular mechanisms on the growth performance of *T. ovatus*.

## 1. Introduction

The gut microbiota refers to a vast array of microorganisms residing in an animal’s intestines, relying on the host’s intestinal system and aiding in the execution of several physiological and biochemical processes [1,2]. The gut microbiota not only governs the health of the host [3,4], but also serves as a crucial link between nutrition and host physiology [5,6]. The gut microbiota governs numerous metabolic processes in the host, such as energy, glucose, lipid and protein metabolism [6,7]. Due to its high metabolic capacity, the gut microbiota plays an important role in the digestion and absorption of nutrients and can influence the efficiency of energy acquisition from the diet and the host’s energy storage [8,9,10,11]. 

Golden pompano, *Trachinotus ovatus*, a widely distributed tropical marine fish, is economically an important member of the Carangidae family due to its delectable flesh. In the past decade, *T. ovatus* has been extensively cultivated in the South China Sea region, including China, Malaysia, Indonesia, and Singapore [12]. The annual production of *T. ovatus* has reached 250 kilo metric tons in 2023 in China. 

Proteins and lipids are two of the more expensive components in fish feeds. Therefore, improving the absorption and utilization of proteins and lipids is not only beneficial to the health and growth of fish, but also helps to reduce the cost of aquaculture. Consequently, employing gut microbiota as a means to enhance the efficacy of protein and fat utilization in aquatic species has become a feasible approach. However, the contribution of the gut microbiota of *T. ovatus* to nutrients and its effect on growth performance are not well-established.

Germ-free animal models have been successfully used to study the succession and role of the gut microbiota in mammals [13,14]. However, germ-free Scleractinia models are difficult to obtain. For example, the lifespan of germ-free zebrafish is too short to readily identify the phenotypic and metabolic characteristics of different organs [15]. Therefore, the use of antibiotic-treated animals to explore the succession and function of the gut microbiota is another popular approach [14,16]. It is convenient to detect the influence of gut microbiota and identify the underlying molecular mechanisms by using antibiotic-treated animals [17].

The objective of this study was to investigate the role of the gut microbiota in improving the growth performance of *T. ovatus* and its functional mechanism by comparing the differences in growth performance, diversity and composition of the gut microbiota, and differences in the metabolite enrichment and gene expression between an antibiotic-treated model and a control group of *T. ovatus*.

## 2. Results

### 2.1. Construction of the Antibiotic-Treated T. ovatus Model

Germ-free *T. ovatus* is difficult to obtain; therefore, in this study, we constructed an antibiotic-treated *T. ovatus* model to explore the role of the gut microbiota. After two days of antibiotic treatment, the total quantity of bacteria in the intestines of TB group decreased to about 2.1 orders of magnitude, which was maintained until the end of the antibiotic treatment, while the control group was basically maintained at about 7.5 orders of magnitude (Supplementary Figure S1A). This result suggests that antibiotics, while not completely eliminating the gut microbiota, can greatly deplete the microbes in the intestines. This result was confirmed by qPCR analysis using universal primers and internal reference primers for total intestinal bacterial genes (Supplementary Figure S1B). Moreover, the survival rate during antibiotic treatment was 100% in all groups.

In the subsequent culture experiments, no differences were found between the antibiotic-treated model and the control *T. ovatus* in terms of vigor and food intake.

### 2.2. Differences in the Growth Performance of Antibiotic-Treated and Control T. ovatus

Comparative analyses showed that the growth performance of TB group was significantly lower than that of the control group (CB). All the indexes, including body weight, body length, body thickness, body depth, WGR and SGR were significantly higher in the CB than in the TB group. However, the FCR was significantly lower than in the TB group (Table 1). These results suggested an important positive effect of gut microbial diversity and stabilization on the growth performance of *T. ovatus*.

### 2.3. Differences in the Gut Microbiota of Antibiotic-Treated and Control T. ovatus

**Figure 1:**
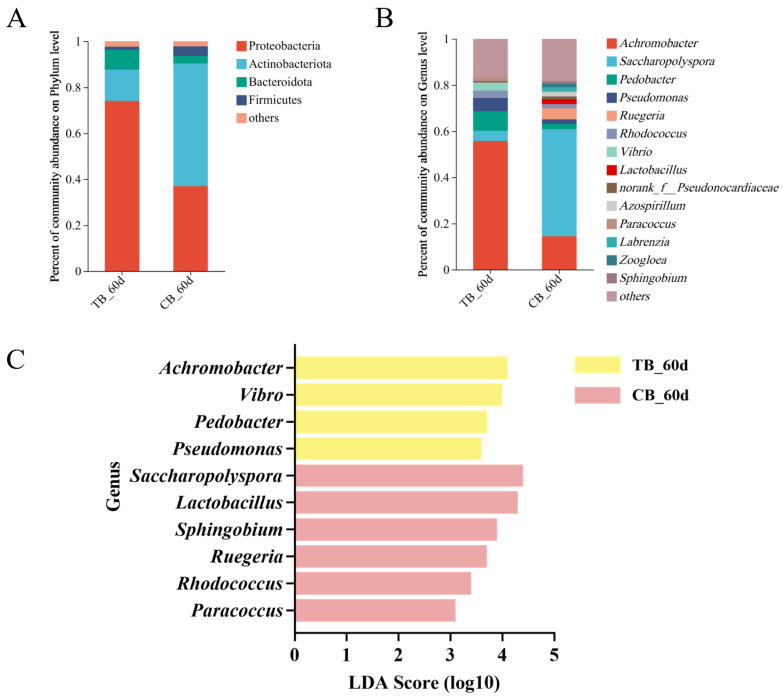
Gut microbiota differences in the response of antibiotic-treated and control *T. ovatus* to the same diets: (**A**,**B**) average relative abundance of dominant bacterial phylum and genus, in which species with a relative abundance of less than 0.01 were classified as others; and (**C**) LEfSe analysis of differential microbials in CB vs TB. Only taxa with a significant LDA Score value > 3 are shown. Larger LDA scores represent a greater effect of species abundance on the differential effect.

Our aim was to investigate the mechanisms by which growth performance, diversity and composition of gut microbiota were influenced in the TB and CB groups. The alpha diversity by the Shannon, Ace and PD indices was significantly higher in the CB group compared to the TB group (Table 2). First, the gut microbiota had similar species composition at the phylum level, but with markedly different proportions. There was relatively less Proteobacteria but more Actinobacteriota in CB; values for Bacteroidota/Firmicutes were higher in the TB group than in the CB group (Figure 1A). At the genus level, differences were also revealed (Figure 1B). The percentages of *Achromobacter, Pedobacter* and *Pseudomonas* in the CB group were significantly lower than in the TB group. By contrast, the percentages of *Saccharopolyspora* and *Ruegeria* in the CB group were significantly higher than that in the TB group. In addition, several genera unique to the CB or TB group were characterized, with *Vibrio* being unique to the TB group and *Lactobacillus*, *Azospirillum*, *Labrenzia*, *Zoogloea* and *Sphingobium* being unique to the CB group (Figure 1B). Following this, LEfSe analysis revealed that some genera contributed considerably to the differences between the two groups. Among these, *Achromobacter*, *Vibrio*, *Pedobacter* and *Pseudomonas* were significantly enriched in TB_60d, while *Saccharopolyspora*, *Lactobacillus*, *Sphingobium*, *Ruegeria*, *Rhodococcus* and *Paracoccus* were significantly enriched in CB_60d (Figure 1C).

### 2.4. Metabolomic Differences in the Antibiotic-Treated and Control Groups

In order to further investigate the mechanism by which differences in gut microbiota led to different growth performance of *T. ovatus*, the intestinal metabolites and their clustering were analyzed in the antibiotic-treated and control groups. The sequencing produced good-quality data for subsequent analysis (Supplementary Table S3).

**Figure 2:**
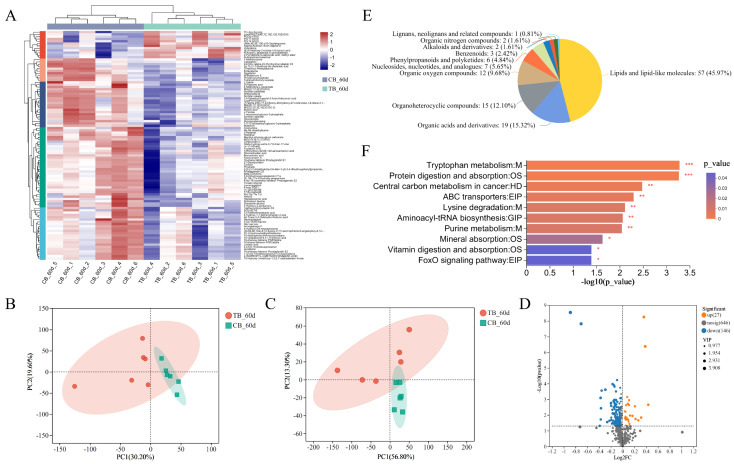
Differences in intestinal metabolite enrichment patterns of *T. ovatus* in TB vs. CB: (**A**) clustering heatmap of metabolites; PCA plots of metabolites of positive (**B**); and negative ions (**C**). (**D**) Volcano plots of the DMs; (**E**) classification of upregulated DMs in the HMDB database; and (**F**) KEGG pathway enrichment of upregulated DMs. * *p* < 0.05; ** *p* < 0.01; *** *p* < 0.001.

The clustering patterns of metabolites were significantly different between the CB and TB groups (Figure 2A). This result was further confirmed by PCA analyses of positive and negative ion metabolites (Figure 2B,C). In addition, metabolite difference analysis showed that 27 metabolites were upregulated, and 146 were downregulated, in the TB vs CB (Figure 2D). 

The HMDB 4.0 database was applied to categorize downregulated DMs. These downregulated DMs were mostly lipids and lipid-like molecules, accounting for up to 45.97% of the total DMs. The remaining categories with higher percentages were organic acids and derivatives (15.32%), organoheterocyclic compounds (12.10%), and organic oxygen compounds (9.68%) (Figure 2E).

KEGG enrichment analyses were performed on downregulated DMs to investigate the potential mechanisms by which these metabolites affect growth performance. These downregulated DMs were most enriched in pathways belonging to amino acid metabolism and digestive system, including tryptophan metabolism, lysine degradation, protein digestion and absorption, mineral absorption, and vitamin digestion and absorption (Figure 2F).

The above results showed that the antibiotic-treated group had suppressed intestinal metabolism and also had lower functions in the digestive system, amino acid metabolism, translation, and signal transduction than the control group.

### 2.5. Differences in Gene Expression between Antibiotic-Treated and Control T. ovatus

To further understand the mechanisms by which host–microbiome interactions affect growth traits, expressional differences of intestinal genes between the TB and CB groups were analysis. A total of 128,934,714 raw reads were generated after up-sequencing of the extracted RNA, and 127,129,622 clean reads remained after QC, with mean values of 97.15% and 92.10% for Q20 and Q30, respectively. Comparing clean reads to the genome of *T. ovatus*, the mean value of the total comparison rate was 93.15% (Supplementary Table S2). These data indicate that the data generated by sequencing is of good quality, is a reliable source, and can meet the subsequent analysis requirements.

**Figure 3:**
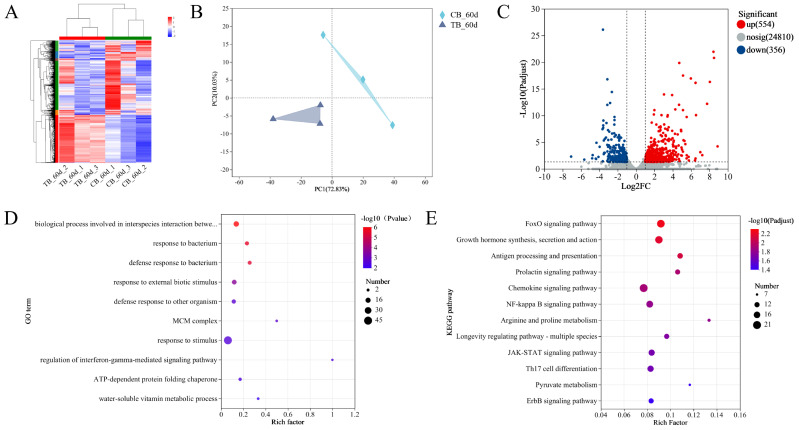
Differences in intestinal gene expression patterns of *T. ovatus* in TB vs. CB: (**A**) clustering heatmap of genes; (**B**) PCA plot of genes in TB vs CB; (**C**) Volcano plots of the DEGs; (**D**) GO term enrichment of upregulated DEGs. The results of the top 10 enrichments in order of *p* value from small to large; and (**E**) KEGG pathway enrichment of upregulated DEGs. The results of the top 12 enrichments in order of *p* adjust from small to large.

The investigation identified different patterns of gene expression in groups TB and CB (Figure 3A,B). A total of 910 DEGs were found in the intestine of TB compared to the CB group, of which 554 were upregulated and 356 were downregulated (Figure 3C). 

The downregulated DEGs were annotated by GO enrichment analysis. Of these, the biological process involved in interspecies interactions between organisms, response to bacterium, defense response to bacterium, and the response to external biotic stimulus were the dominant subcategories in biological processes; the cell and MCM complex were the dominant subcategories in the cellular components; and the ATP-dependent protein folding chaperone was the dominant subcategory in molecular functions (Figure 3D). All the downregulated DEGs were further analyzed by KEGG pathway analysis. The pathways involved in signal transduction were the FoxO signaling pathway, NF-kappa B signaling pathway, and JAK-STAT signaling pathway. The pathways related to the immune system included antigen-processing and presentation, the chemokine signaling pathway, and Th17 cell differentiation. In addition, some of the downregulated DEGs were highly enriched in growth hormone synthesis, secretion, and action (Figure 3E).

The top two enriched pathways of the KEGG pathway were those associated with cell and host growth, including the FoxO signaling pathway, and growth hormone synthesis, secretion, and action. Combined with gene expression analysis, we screened 10 genes with large expression differences in these two pathways as candidate genes, including *ghr*, *jak2*, *stat3*, *socs1*, *cdkn1a*, *egfr*, *shc1*, *sos1*, *kras* and *mapk1* (Supplementary Table S1).

### 2.6. Expression Analysis of Key Genes

**Figure 4:**
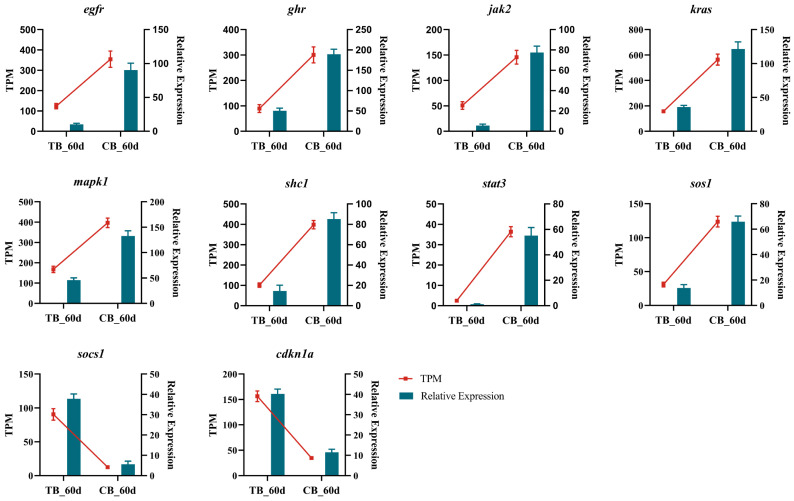
qPCR validation of transcriptome data. Bar graphs are qPCR data, showing the mean ± SE (*n* = 3) of relative expression, and the samples used are three samples from transcriptome sequencing. The line graph shows the transcriptome data.

We analyzed ten genes from the transcriptome using qPCR to assess their expression in both groups and validate the transcriptome results. The qPCR results demonstrated that the gene expression pattern matched that of the transcriptome data, suggesting the reliability of the transcriptome sequencing and analysis (Figure 4). The genes *socs1* and *cdkn1a* (*p21*) were strongly expressed in the TB group and lowly expressed in the CB group, while the other genes showed the opposite pattern, being highly expressed in the CB group, and lowly expressed in the TB group.

**Figure 5:**
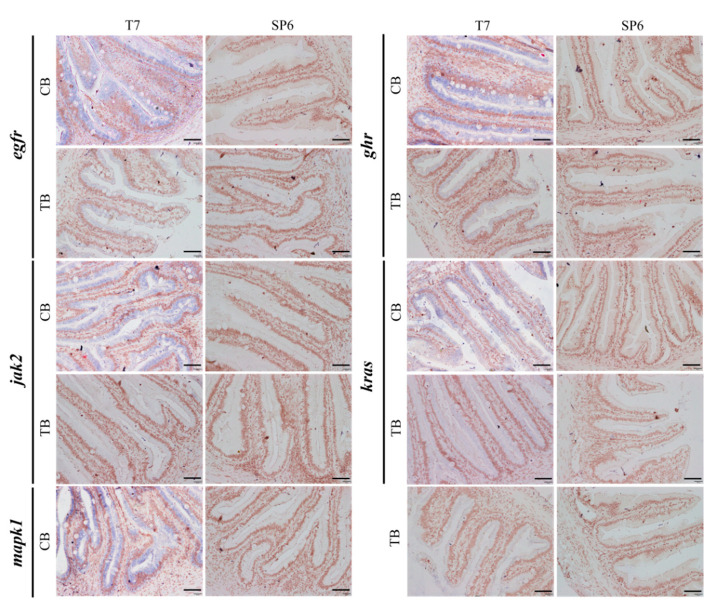
Localization of key genes in the intestine of group TB and group CB *T. ovatus*. Scale bars = 40 μm.

In order to further verify the reliability of these key genes and to validate the expression locations of these genes, we selected five key genes (*egfr*, *ghr*, *jak2*, *kras*, *mapk1*) for in situ hybridization experiments. The experimental results showed that all five genes, *egfr*, *ghr*, *jak2*, *kras* and *mapk1*, had in situ hybridization signals in the columnar epithelial cells of the intestinal villi of both the TB and CB groups, but there was a significant difference in the intensity of signals (Figure 5). The signal intensities of the CB group were significantly higher than those of the TB group, which indicated that these genes were highly expressed in the CB group and lowly expressed in the TB group.

### 2.7. Correlation between Intestinal Bacteria and the DEGs and DMs

**Figure 6:**
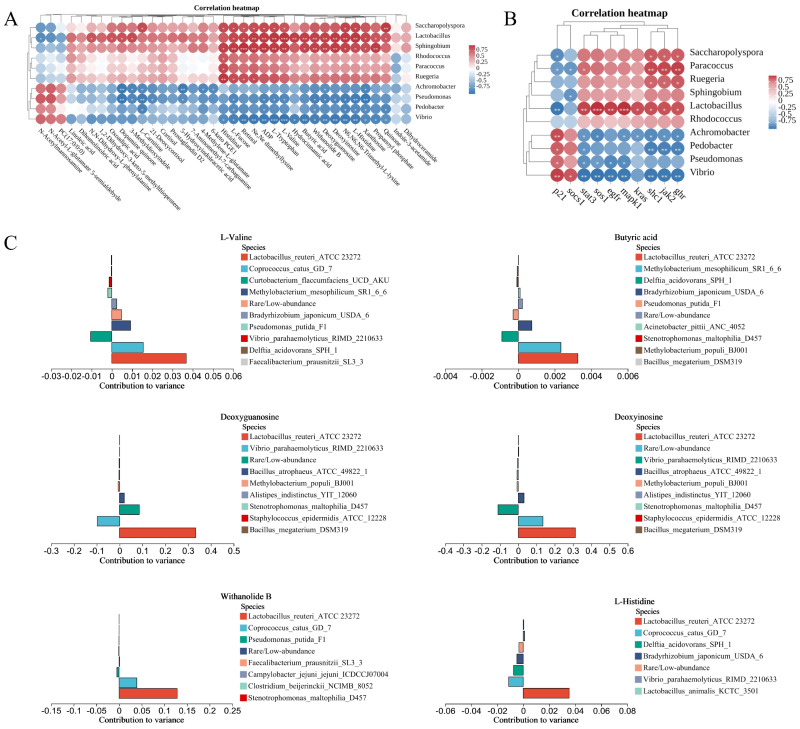
The correlation between intestinal bacteria and DMs and DEGs: (**A**) Pearson correlation analysis between intestinal bacteria and DMs; (**B**) Pearson correlation analysis between intestinal bacteria and DEGs. The correlation coefficient is represented by different colors (red, positive correlation; blue, negative correlation). *: *p* < 0.05; **: *p* < 0.01; ***: *p* < 0.001; and (**C**) histogram of bacteria contribution to metabolites by MIMOSA2 model analysis. The figure shows the six metabolites that are most affected by bacteria and the top 10 (or all of them if there are less than 10) microbes contributing to theses metabolites. The horizontal coordinate is the contribution to variance, values greater than 0 mean that these bacteria contribute to the production of the metabolite, and values less than 0 mean that these bacteria consume the metabolite.

Correlation analyses were performed to reveal the relationships between host growth and intestinal bacteria, metabolites, gene expression. We selected DMs that could be annotated in the KEGG database and the 10 bacteria with more significant differences of TB_60d vs. CB_60d (Figure 1C) for Pearson correlation analysis. The results showed that six of the bacteria highly enriched in the CB group, including *Saccharopolyspora*, *Lactobacillus*, *Sphingobium*, *Ruegeria*, *Rhodococcus* and *Paracoccus*, were positively correlated with most of the metabolites and negatively correlated with only three metabolites. The remaining four bacteria (*Achromobacter*, *Vibrio*, *Pedobacter* and *Pseudomonas*) had the opposite result (Figure 6A). The two genera most strongly correlated with metabolites were *Lactobacillus* and *Vibrio*, with *Lactobacillus* being positively and *Vibrio* negatively associated with most metabolites (Figure 6A). The composition of the gut microbiota and the concentrations of metabolites were correlated by the MIMOSA2 model. The results showed that the six metabolites on which the intestinal bacteria had the greatest influence were L-Valine, Butyric acid, Deoxyguanosine, Deoxyinosine, Withanolide B and L-Histidine (Figure 6C). It is worth noting that *Lactobacillus_reuteri_ATCC_23272* has potential promoters of all six metabolites (Figure 6C).

The correlations between the 10 candidate genes (Supplementary Table S1) associated with cell and host growth obtained from the transcriptome analysis and the 10 bacterial species were then analyzed by Pearson correlation analysis (Figure 6B). The results showed that *Saccharopolyspora*, *Lactobacillus*, *Sphingobium*, *Ruegeria*, *Rhodococcus* and *Paracoccus* were positively correlated with *ghr*, *jak2*, *stat3*, *egfr*, *shc1*, *sos1*, *kras*, and *mapk1* and negatively correlated with *socs1* and *cdkn1a*. The other four bacteria (*Achromobacter*, *Vibrio*, *Pedobacter* and *Pseudomonas*) exhibited the opposite result. Interestingly, *ghr*, *jak2*, *stat3*, *egfr*, *shc1*, *sos1*, *kras* and *mapk1* were highly expressed in the CB group, while *socs1* and *cdkn1a* were highly expressed in the TB group. In other words, the six microbial genera that were highly enriched in the CB group may have contributed to the high expression of these eight genes in the CB group.

## 3. Discussion

The gut microbiota is a critical and direct regulator of fish physiology, immunity, metabolism, and health [18]. It can carry out many processes that the host cannot, such as the production or regulation of metabolites, which may act as metabolic substrates or signaling molecules for the host and are important for host metabolism and health [19]. This study investigated the role of gut microbiota in enhancing host growth performance and its mechanism of action by analyzing differences in microbial composition, metabolite enrichment and gene expression between an antibiotic-treated model and a control *T. ovatus* under the same high-nutrient dietary condition.

In this study, we successfully constructed the antibiotic-treated *T. ovatus* model. The role of gut microbiota on the growth of *T. ovatus* and its mechanisms could then be explored subsequently by comparing the differences in growth performance, gut microbiota, intestinal metabolites, and expression of intestinal genes between the antibiotic-treated model and the control *T. ovatus*.

After 30 days of feeding, we observed significant differences between antibiotic-treated (TB) and control (CB) groups in various growth performance indicators (Table 2). The growth performance of the CB group was significantly higher than that of the TB group, demonstrating the important role of gut microbiota in improving growth performance. Meanwhile, we discovered that the diversity and composition of the gut microbiota of the CB and TB groups remained significantly different after 30 days of feeding. Research has demonstrated that Rhodobacteraceae, particularly *Ruegeria*, can improve the structure and stability of the gut microbiota, and have a significant impact on the aquaculture performance of Pacific white shrimp [20]. *Rhodococcus* is capable of producing lipids from a wide range of carbon sources. Additionally, it has a distinctive metabolic pathway for breaking down alkanes and aromatic hydrocarbons, allowing it to utilize not only common carbon sources like glucose and fructose, but also alkanes and aromatic compounds [21,22,23]. Although the role of *Rhodococcus* in the host intestine is not well-understood, it can only be treated as a potentially beneficial microbe. All these studies showed that the stability and percentage of beneficial bacteria of the gut microbiota in the CB group was higher than that of the recolonized gut microbiota in the TB group.

The intestinal metabolome also responds differently to the different gut microbiota of TB and CB. These DMs were much more abundant in the CB group than in the TB group, a finding that somewhat supports the important function of gut microbiota in metabolism. The downregulated DMs in the TB group compared to the CB group were mainly lipids and lipid-like molecules and organic acids and derivatives. It has been shown that the gut microbiota can react with fatty acid double bonds to produce metabolites that cannot be synthesized in mammals, such as linoleic acid [24,25]. The gut microbiota affects the lipid composition of host tissues and serum and downregulates cholesterol biosynthesis [26,27]. It also has a significant impact on protein metabolism and absorption breaking down complex subunits and altering the metabolic machinery of the host cell to facilitate protein or amino acid digestion and absorption [28,29]. Besides, the gut microbiota can promote the de novo synthesis of essential amino acids [30]. The KEGG enrichment analysis revealed that downregulated DMs in TB and CB were predominantly associated with pathways involved in nutrition metabolism, absorption, and transport. Based on the findings and observations presented above, it can be concluded that a well-established gut microbiota not only directly participates in the digestion and metabolism of nutrients but also facilitates the digestion, absorption and transport of substances in the intestine of *T. ovatus* by modulating relevant pathways through the metabolites produced.

The Pearson correlation analysis of the DMs and the 10 bacterial species showed a positive correlation between microbes with the same trend and DMs and a negative correlation between microbes with the opposite trend and DMs (Figure 6A). Two of the genera showing the strongest correlations with metabolites were *Lactobacillus* and *Vibrio*. *Lactobacillus* was more enriched in the CB group. It can synthesize organic acids and other nutrients, through which it regulates the host’s gut microbiota and promotes the synthesis of digestive enzymes and minerals. This is conducive to the absorption and utilization of the diet and nutrient metabolism, thus promoting the healthy growth of the host [31,32,33]. *Vibrio* spp. are predominantly pathogenic bacteria that can present a substantial risk to the health of humans and marine animals [34,35,36]. The DMs are enriched in several pathways related to nutrient metabolism, absorption and signal transduction. This indicates that *Lactobacillus* promotes these pathways while *Vibrio* inhibits them. Additionally, the MIMOSA2 model analysis revealed that six DMs in the CB group were closely associated with specific gut microbiota: L-Valine, Butyric acid, Deoxyguanosine, Deoxyinosine, Withanolide B and L-Histidine. Valine and histidine are essential nutrients for fish. They are involved in a variety of physiological processes, including protein synthesis, lipolysis and immunity, and promote the secretion of growth hormone to promote growth [37,38]. Butyric acid is a short-chain fatty acid that plays a crucial role in maintaining the gut barrier, immunity and host growth [39,40]. The synthesis of these metabolites is also mostly associated with *Lactobacillus_reuteri_ATCC_23272*. These results confirm that *Lactobacillus* is essential for the production of metabolites capable of promoting host growth, which corroborates the ability of gut microbiota to increase the dietary utilization efficiency and thus promote the growth of *T. ovatus*.

To further enhance our understanding of the mechanisms by which gut microbiota influences host growth performance, we performed transcriptome analyses of the intestines of the *T. ovatus*. The downregulated DEGs by TB compared to CB in GO enrichment were primarily correlated with terms related to responses to external organisms, bacteria, and interspecies interactions between organisms. The KEGG pathway enriched for these downregulated DEGs is also mostly associated with the immune system and signal transduction. These results indicated that the absence of gut microbiota weakens the intestine’s ability to respond to bacteria and affects signaling between intestinal tissues and the outside envrierenment, which in turn affects the physiological functions and immunity of the intestine. Taken together, the DMs and DEGs in both CB vs CA and TB vs CB were frequently enriched for several KEGG pathways, including MAPK signaling pathway, FoxO signaling pathway and growth hormone synthesis, secretion and action. Analysis of DEGs in these pathways revealed several significantly different genes associated with the Ras/ERK signaling pathway. The Ras/ERK pathway is an important enzyme-protein signaling system in the MAPK family, which is involved in the regulation of a variety of physiological activities, including cell proliferation and differentiation [41]. Ras can be activated by stimulation of the membrane receptor bound to the GRB2 (growth factor receptor binding protein 2)/SOS (son of sevenless) complex [42]. Activated Ras directly interacts with and activates Raf, and activated Raf continues to activate MEK and ERK by regulating various transcription factors involved in gene transcription, protein synthesis, cell differentiation, and proliferation [43,44]. EGFR (epidermal growth factor receptor) tyrosine phosphorylation activates Ras and transduces signals from the cell surface to the nucleus via the Ras/ERK pathway, mediating DNA synthesis and cell proliferation effects. The JAK/STAT signaling pathway is one of the major intracellular signal transduction pathways. In addition, GHR can also activate the Ras/ERK pathway by activating JAK2. It is worth mentioning that the genes we selected for subsequent analyses are also mostly involved in these pathways.

Microorganisms upregulated in the CB group were positively correlated with upregulated genes and negatively correlated with downregulated genes, a result consistent with that in the TB group. Similar to the results of the correlation analysis of DMs with microbes, *Lactobacillus* and *Vibrio* were the two genera most highly correlated with these DEGs. *Lactobacillus* is positively correlated with most of the DEGs in Ras/ERK signaling pathway, indicating that *Lactobacillus* may promote the growth of *T. ovatus* by promoting the expression of these genes. *Vibrio* was positively correlated with the repressors (*socs1* and *cdkn1a*) in Ras/ERK signaling pathway, indicating that *Vibrio* may negatively regulate the growth of *T. ovatus* by promoting the expression of these genes.

Regrettably, this study has several limitations. First, this study was limited only to intestines, and did not involve the brain, liver, and muscle. The hypothalamic–pituitary–somatotropic (HPS) axis plays a crucial role in the endocrine regulation of growth in vertebrates. The hypothalamus secretes growth hormone-releasing hormone (GHRH) and somatostatin (SS) to regulate the synthesis and secretion of growth hormone (GH), which binds to the growth hormone receptor (GHR) on target cell via the blood circulation [45,46]. This combination stimulates liver secretion of insulin-like growth factor 1 (IGF1), which later binds to the IGF1 receptor (IGF1R) on target cells and ultimately stimulates cell proliferation and differentiation [47,48]. Although the present study involves the study of GHR, neither the release of GH nor the action of IGF1 is addressed. Furthermore, while the correlation analyses suggested that *Lactobacillus* may contribute to the improved growth performance of *T. ovatus*, there was a lack of support through additional feeding experiments. Thus, a very comprehensive account of the mechanisms by which gut microbiota assists dietary co-action to enhance the growth performance of *T. ovatus* requires further research.

## 4. Materials and Methods

### 4.1. Experimental Design and Sample Collection

Healthy 30 dph (day post-hatching) *T. ovatus* with an average body weight of 1.48 ± 0.14 g and an average total length of 2.43 ± 0.21 cm were randomly obtained from the culture ponds of Hainan Chenhai Aquatic Co. The fish were acclimated in circulating aerated seawater (salinity 30‰, pH 8.1, temperature 26 ± 1 °C) for 3 days before the feeding experiment. The fish were offered commercial feed at 2% of their body weight twice daily. The fish were divided into two experimental groups: one group was the antibiotic-treated *T. ovatus* model (TB), and the other group was the control group (CB). Each group was reared in two separate circular tanks (Φ = 0.8 m, h = 1 m) containing 500 L of seawater with 200 individuals per tank.

Differential dietary feeding experiment lasted 60 days. Individuals in the experimental groups at were sampled at 60 dph (i.e., after 30 days of differential dietary feeding) for intestinal contents and tissue, respectively. Feeding was stopped 24 h before sampling for all experimental individuals. Six individuals were randomly selected from each tank as replicate samples. All samples were immediately frozen in liquid nitrogen and stored at −80 °C until RNA isolation for sequencing.

### 4.2. Antibiotic-Treated T. ovatus Model

Germ-free ovomucoid pomace was difficult to obtain, so we chose to construct an antibiotic treatment model for subsequent studies. Based on previous relevant studies on zebrafish, vancomycin, metronidazole, neomycin sulfate, and ampicillin were used to deplete intestinal bacteria in *T. ovatus* [16,49]. The four antibiotics were added to both the rearing water and the diets. Concentrations of metronidazole, ampicillin, neomycin sulfate and vancomycin were 0.5, 0.5, 0.5 and 0.01 mg/mL in rearing water, and 5, 5, 2, and 1 mg/g in the diets, respectively. The rearing water was changed twice a day and the antibiotics were replenished to maintain the concentrations after the water change. Antibiotic treatment lasted for four days. The quantity of intestinal microorganisms was determined daily by smearing during the antibiotic treatment. The total quantity of bacteria in the intestine needs to be determined on a daily basis during the antibiotic treatment. The intestinal contents of three individuals were randomly collected daily from each group. After being homogenized with 1 mL of PBS, 100 µL of the homogenized mixture from each sample was plated on LB plates for bacterial counts. At the end of the antibiotic treatment, the microbial 16S rRNA gene was detected using the universal primers Uni331-F/Uni797-R (uni331-F: 5′-TCCTACGGGGAGGCAGCAGT-3′; uni797-R: 5′-GGACTACCAGGTATCTATCCTGTT-3′) [50] and the internal reference primer β-actin (β-actin-F: 5′-TCGATCATGAAGTGCGATGT-3′; β-actin-R: 5′-ATCAGCAATACCGGGGGTACA-3′) were used to quantify the total quantity of the bacteria in the intestine and to verify the efficacy of the antibiotic treatment.

At the end of the antibiotic treatment, the antibiotic-treated model, and the control *T. ovatus*, were subjected to the same diet and the same environment at the same time in the culture experiment.

### 4.3. Growth Performance Analyses

At the end of the trial, ten individuals were randomly selected from each tank as replicate samples; body weight, total length, body thickness, and body depth were measured. Metrics assessed for growth performance included weight gain rate (WGR), specific growth rate (SGR), and feed conversion ratio (FCR). The calculations were as follows: WGR = 100 (W_t_ − W_0_)/W_0_; SGR = 100 (lnW_t_ − lnW_0_)/t; and FCR = FI/(W_t_ − W_0_). W_t_ is the body weight of fish at day t, W_0_ is the initial weight of fish, t is the duration of feeding (in days), and FI is the feed intake (g).

### 4.4. Intestinal Microbiome Analysis

The total genomic DNA of the microbes from the intestine was extracted. Then, the V4 region of the bacterial 16S rDNA gene was amplified using a pair of primers, 515F (5′-GTGCCAGCMGCCGCGG-3′) and 806R (5′-GGACTACHVGGGTWTCTAAT-3′). Operational taxonomic units (OTUs) with a similarity greater than or equal to 97% were clustered and analyzed for classification [51,52]. Bioinformatic analysis of the gut microbiota was done using Majorbio Cloud (https://cloud.majorbio.com, accessed on 1 March 2023). Three metrics were used to calculate alpha diversity, including Shannon, Ace, and PD indices. Beta diversity was assessed through principal coordinate analysis (PCoA) plots based on Bray–Curtis metrics. Microbial composition was analyzed at the phylum and genus levels. Linear discriminant analysis effect size (LEfSe) was successively performed using the Kruskal–Wallis rank sum test, the Wilcoxon rank sum test, and linear discriminant analysis (LDA) to obtain significantly different species [53].

### 4.5. Intestinal Metabolomics Analysis

Intestinal content samples from six replicate individuals per group were used for the metabolomics analysis. The LC-MS/MS analysis of sample was conducted on a Thermo UHPLC-Q Exactive HF-X system equipped with an ACQUITY HSS T3 column (100 mm × 2.1 mm i.d., 1.8 μm; Waters, Milford, MA, USA) at Majorbio Bio-Pharm Technology Co., Ltd. (Shanghai, China). The optimal conditions were set as follows: source temperature at 425 °C; sheath gas flow rate at 50 arb; aux gas flow rate at 13 arb; ion-spray voltage floating (ISVF) at −3500 V in negative mode and 3500 V in positive mode, respectively; normalized collision energy, 20-40-60 V rolling for MS/MS. Full MS resolution was 60,000, and MS/MS resolution was 7500. Data acquisition was performed with the Data Dependent Acquisition (DDA) mode. The detection was carried out over a mass range of 70–1050 *m*/*z*.

The pretreatment of LC/MS raw data was performed by Progenesis QI 3.0 (Waters Corporation, Milford, MA, USA) software, and a three-dimensional data matrix in CSV format was exported. Then, the R package “ropls” (Version 1.6.2) was used to perform principal component analysis (PCA) and orthogonal least partial squares discriminant analysis (OPLS-DA), and 7-cycle interactive validation evaluating the stability of the model. The metabolites with VIP > 1, *p* < 0.05 were determined as significantly different metabolites based on the variable importance in the projection (VIP) obtained by the OPLS-DA model and the *p*-value generated by student’s *t* test. The data were analyzed online (https://www.majorbio.com). All metabolites were classified according to KEGG and the Human Metabolome Database (HMDB). Differential metabolites (DMs) of CA vs. CB and TB vs. CB were identified. Statistical analyses of KEGG enrichments were set at *p* values < 0.05.

### 4.6. Intestinal Transcriptomic Analysis

Intestinal tissue samples of three replicate individuals from each group were used for transcriptomic analysis. Three libraries were constructed for each of the TB and CB groups, and a total of six libraries were constructed. Total RNA was extracted from the intestine using TRIzol^®^ reagent (Invitrogen, Waltham, MA, USA). RNA purification, reverse transcription, library construction and sequencing were performed at Shanghai Majorbio Bio-pharm Biotechnology Co., Ltd. (Shanghai, China) according to the manufacturer’s instructions (Illumina, San Diego, CA, USA). After quantified by Qubit 4.0, paired-end RNA-seq sequencing library was sequenced with the NovaSeq 6000 sequencer (2 × 150 bp read length). The raw paired-end reads were trimmed and quality-controlled by fastp [54] with default parameters. Then clean reads were separately aligned to a reference genome with orientation mode using HISAT2-2.2.1 [55] software. The mapped reads of each sample were assembled by StringTie [56] in a reference-based approach.

Differentially expressed genes (DEGs) were defined as genes with |log_2_ (FoldChange)| > 2 and a false discovery rate (FDR) < 0.05. Statistical analyses of GO were set at *p* value < 0.05 and KEGG enrichments were set at BH-corrected *p* adjust <0.05.

### 4.7. Expression and Localization Analysis of Key Genes

Analysis of the expression amount and location of key genes using qPCR and in situ hybridization (ISH). The steps are quoted from previous methods in our laboratory [57]. The primers used for qPCR and ISH are detailed in Table 3 and Table 4.

### 4.8. Correlation Analysis

Gut microbiota species composition and metabolite concentrations were correlated through the MIMOSA model. The MIMOSA model links species composition and metabolite concentrations by predicting the community-wide metabolite potential, constructing a metabolic model to predict the effect of community composition on metabolite concentrations, and assessing whether this prediction is consistent with measured metabolomic profiles [58]. Pearson correlation analysis was used to reveal correlations between intestinal bacteria and host intestinal DEGs and DMs.

### 4.9. Statistical Analysis

The data results of this study had at least three replicates and were expressed as mean ± SE. The data were analyzed using GraphPad Prism. Differences between groups were analyzed using one-way ANOVA. *p* < 0.05 was regarded as statistically significant, *p* < 0.01 was regarded as highly significant, and *p* < 0.001 was regarded as extremely significant.

## 5. Conclusions

In summary, we discovered that the gut microbiota can improve its own composition by mediating the colonization of some probiotics represented by *Lactobacillus* in the intestine, improving host metabolic efficiency in proteins and lipids, and also influencing the expression of genes in signaling pathways related to cell proliferation, which together contribute to the improved growth performance of *T. ovatus*. These results revealed the important role and functional mechanism of gut microbiota on the growth of *T. ovatus*, providing new insights into the study of gut microbiota in aquatic organisms, and a theoretical basis for the subsequent study of functional feed additives to improve growth performance. Overall, fish microbiota research can provide insights into the mechanism of host–microbiota interactions and develop novel tools to promote fish health and the sustainability of the aquaculture industry.

## Figures and Tables

**Table 1:** Differences in growth performance of *T. ovatus* after differential dietary feeding.

Group	TB	CB
W_0_ (g)	1.33 ± 0.09	1.33 ± 0.09
W_t_/Body weight (g)	2.40 ± 0.13 ^a^	4.33 ± 0.21 ^c^
Body length (cm)	6.15 ± 0.41 ^a^	7.91 ± 0.55 ^c^
Body thickness (cm)	1.13 ± 0.04 ^a^	1.25 ± 0.07 ^b^
Body depth (cm)	2.05 ± 0.11 ^a^	2.32 ± 0.08 ^b^
WGR (%)	80.45 ± 3.89 ^a^	225.56 ± 15.37 ^b^
SGR (%/d)	1.96 ± 0.10 ^a^	3.93 ± 0.25 ^c^
FCR (%)	1.53 ± 0.04 ^a^	1.40 ± 0.05 ^b^

Note: W_t_ is the weight of fish at day t, W_0_ is the initial weight of fish, t is the duration of feeding (in days). WGR, weight gain rate; SGR, specific growth rate; FCR, feed conversion ratio. Values are represented as the mean ± SE (*n* = 10). Different superscript letters (a, b, c) indicate differences between groups, adjacent letters indicate significant differences (*p* < 0.05), and intervening letters indicate highly significant differences (*p* < 0.01).

**Table 2:** Alpha diversity of the gut microbiota of *T. ovatus* after differential dietary feeding.

Group	Shannon	Ace	PD
CB_60d	2.42 ± 0.32 ^a^	704.28 ± 107.45 ^a^	24.89 ± 3.96 ^a^
TB_60d	0.81 ± 0.09 ^c^	237.62 ± 67.67 ^c^	8.54 ± 2.57 ^c^

Note: Values represent the mean ± SE (*n* = 3). Different superscript letters (a, c) indicate highly significant differences (*p* < 0.01).

**Table 3:** The primers used for qPCR.

Gene Name	Primers	Sequences (5′-3′)
*ghr*	F-ghr-q	ACCGTCACTTCAGCAACTTCCA
	R-ghr-q	CCACAGGCGTCAGGTCACAT
*jak2*	F-jak2-q	CCAGAGGTTGCGTCTCGTTGA
	R-jak2-q	CAGCGGCTTGTCTCGTGTCT
*shc1*	F-shc1-q	GTGGAGGTGCTACAGTCAATGC
	R-shc1-q	ACGTCAGACAGCGAGAGGAAG
*kras*	F-kras-q	TGCCATCAACAACACCAAGTCC
	R-kras-q	GTGCTCAGGTCGCTCTTATTCC
*mapk1*	F-mapk1-q	GGCACGGCACATTGACAACA
	R-mapk1-q	CGCAGGATCTGGTAGAGGAAGT
*egfr*	F-egfr-q	CAGAACGGAGTCTCGGATGTGA
	R-egfr-q	TTGGAGGTGAGCGGGAGGAT
*sos1*	F-sos1-q	AGGTAAGGCGATGAGGAAGTGG
	R-sos1-q	TCTGGAAGGTGATGCTGTGACT
*stat3*	F-stat3-q	GGTGTGGCTGGACAACATCATT
	R-stat3-q	CCTCCTTGCTGCTCTCACTGAA
*socs1*	F-socs1-q	CAGCGGTCAGCCTGATGTCT
	R-socs1-q	TGAGCAGAGCGAAGAGTGATGT
*cdkn1a (p21)*	F-cdkn1a-q	CTTCTGCCATCTCTATCCTCCT
	R-cdkn1a-q	TGGTCCTGTGGTTGATGTTGA
*β-actin*	F-β-actin-q	TACGAGCTGCCTGACGGACA
	R-β-actin-q	GGCTGTGATCTCCTTCTGC

Note: F: forward primer; R: reverse primer.

**Table 4:** Primers used for in situ hybridization (ISH).

Gene Name	Primers	Sequences (5′-3′)
*ghr*	F-ghr-ISH	ATTTAGGTGACACTATAGAAGAGGCCGACTACCAAGCCAGGAA
	R-ghr-ISH	TAATACGACTCACTATAGGGAGAGCTGAGGTCCAGAGGAGTTCTT
*jak2*	F-jak2-y	ATTTAGGTGACACTATAGAAGAGAGCCTACGCATCGACTTGT
	R-jak2-y	TAATACGACTCACTATAGGGAGATTCAGCAACATCACCTTCAACA
*kras*	F-kras-ISH	ATTTAGGTGACACTATAGAAGAGGAGACCAACCGCAGCAACAAG
	R-kras-ISH	TAATACGACTCACTATAGGGAGACGGACTCTGCACATTGGATGGA
*mapk1*	F-mapk1-ISH	ATTTAGGTGACACTATAGAAGAGGGCACGGCACATTGACAACA
	R-mapk1-ISH	TAATACGACTCACTATAGGGAGATTGAGGTCGCAGGTGGTGTT
*egfr*	F-egfr-ISH	ATTTAGGTGACACTATAGAAGAGCTCACCTCCAACGGCACAGT
	R-egfr-ISH	TAATACGACTCACTATAGGGAGAAACGGAAAGCTGCACAAAGTGT

Note: F: forward primer; R: reverse primer.

## Data Availability

The data presented in this study are available on request from the corresponding author due to privacy.

## Supplementary Materials

The following supporting information can be downloaded at: https://www.mdpi.com/article/10.3390/ijms25105525/s1.
