# Correction: Kong et al. A Well-Established Gut Microbiota Enhances the Efficiency of Nutrient Metabolism and Improves the Growth Performance of *Trachinotus ovatus*. *Int. J. Mol. Sci.* 2024, *25*, 5525

**DOI:** 10.3390/ijms262110317

**Published:** 2025-10-23

**Authors:** Miao Kong, Wendong Zhao, Cong Wang, Jie Qi, Jinxiang Liu, Quanqi Zhang

**Affiliations:** 1 Key Laboratory of Tropical Aquatic Germplasm of Hainan Province, Sanya Oceanographic Institution, Ocean University of China, Sanya 572025, China; km17669309286@163.com (M.K.); wendongzhaoa@163.com (W.Z.); wangcong11011@stu.ouc.edu.cn (C.W.); qijie@ouc.edu.cn (J.Q.); liujinxiang@ouc.edu.cn (J.L.); 2 MOE Key Laboratory of Marine Genetics and Breeding, Ministry of Education, Ocean University of China, Qingdao 266003, China

In the original publication [1], there was a mistake in Figure 5 as published. During the preparation of Figure 5, the image TB_*egfr*_SP6 was inadvertently reused as CB_*jak2*_SP6, resulting in duplicate image presentation, whereas the correct CB_*jak2*_SP6 figure was inadvertently omitted. The previously misused TB_*egfr*_SP6 image has been replaced with the correct CB_*jak2*_SP6 figure in the revised version. The corrected Figure 5 appears below.

The authors state that the scientific conclusions are unaffected. This correction was approved by the Academic Editor. The original publication has also been updated.

## Figures and Tables

**Figure 5:**
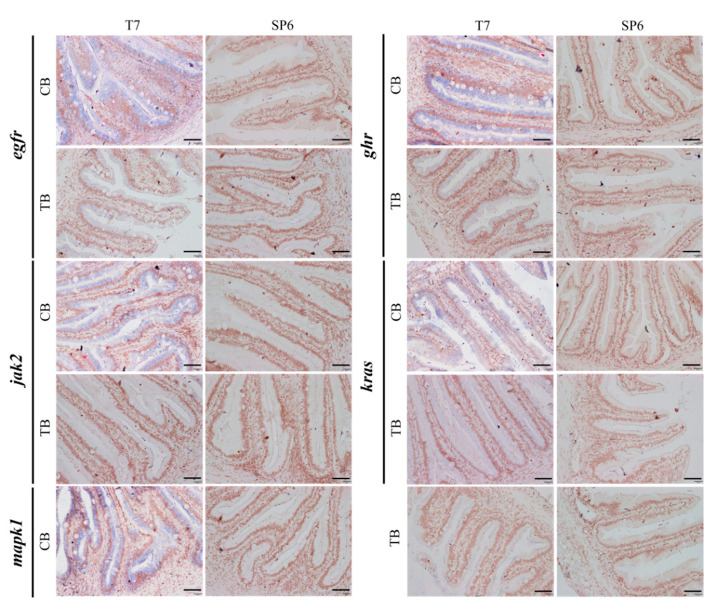
Localization of key genes in the intestine of group TB and group CB *T. ovatus*. Scale bars = 40 µm.
