# Supplementary material for: A Well-Established Gut Microbiota Enhances the Efficiency of Nutrient Metabolism and Improves the Growth Performance of Trachinotus ovatus
Source: Int J Mol Sci. 2024 May 18;25(10):5525. doi: 10.3390/ijms25105525 (PMC11121967; doi:10.3390/ijms25105525)
Supplement: Supplementary file 1 [file ijms-25-05525-s001.zip › ijms-2981127-supplementary.pdf]

Supplementary Table S1 DEGs associated with cell growth in the intestines of *T. ovatus* in the antibiotic-treated and control groups

Gene name	Function annotation	TB vs CB
socs1	Suppressor of cytokine signaling 1 (SOCS1)	up
cdkn1a	Cyclin-dependent kinase inhibitor 1 (CDKN1A/P21)	up
mapk1	mitogen-activated protein kinase 1 (MAPK1)	down
kras	GTPase KRas (KRAS)	down
sos1	Son of sevenless homolog 1 (SOS1)	down
shc1	SHC-transforming protein 1 (SHC1)	down
jak2	Janus kinase 2 (JAK2)	down
ghr	Growth hormone receptor (GHR)	down
stat3	Signal transducer and activator of transcription 3 (STAT3)	down
egfr	Epidermal growth factor receptor (EGFR)	down

Supplementary Table S2 Quality assessment and comparison rate analysis of transcriptome data

Sample	Raw reads	Clean reads	Clean bases	Q20(%)	Q30(%)	GC content(%)	Total mapped(%)
TB_60d_1	42285646	41585244	6162702317	97.14	92.08	51.98	93.41%
TB_60d_2	46724810	46142572	6820776513	97.17	92.18	50.51	93.14%
TB_60d_3	39924258	39401806	5844522686	97.14	92.05	52.63	92.89%
CB_60d_1	44356266	43690662	6447839647	97.41	92.74	53.78	95.85%
CB_60d_2	44208538	43631108	6422153820	97.44	92.68	54.03	95.18%
CB_60d_3	45358888	44857758	6614827634	97.48	92.85	53.07	95.27%

Supplementary Table S3 Total ion number and identification statistics of metabolites

Ion mode	All peaks	Identified metabolites	Metabolites in Library	Metabolites in KEGG
pos	7192	569	440	197
neg	5272	250	214	92

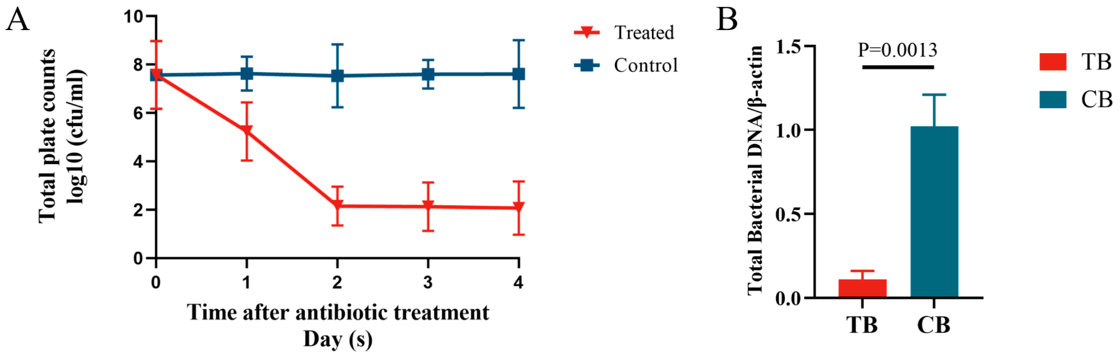

Supplementary Fig. S1 Effect of antibiotic treatment on the intestinal bacterial quantity of *T. ovatus*. (A)Change of bacterial quantity of antibiotic treatment for 4 days. (B) qPCR analysis on the intestine (n = 3) was performed using universal primers for total intestinal bacterial genes. Three biological replicates were performed. Error bars indicate the mean values of SD. Significant differences were determined by the Student's t test, $p = 0.0013$.
